# Functional outcome in intradural extramedullary tumor patients: Case series

**DOI:** 10.1016/j.amsu.2020.04.009

**Published:** 2020-04-24

**Authors:** Fachrisal, Eko Setiawan, Sammy Saleh Alhuraiby

**Affiliations:** a Staff of Department of Orthopaedics & Traumatology, Spine Subdivision, Fatmawati General Hospital, Faculty of Medicine, Universitas Indonesia, Jalan RS Fatmawati No. 1, South Jakarta, Jakarta, 12430, Indonesia; b Resident, Department of Orthopaedics & Traumatology, Cipto Mangunkusumo National Central Hospital and Faculty of Medicine, Universitas Indonesia, Jalan Diponegoro No. 71, Central Jakarta, Jakarta, 10430, Indonesia

**Keywords:** Intradural, Tumor, Outcome, Karnofsky, Tomita

## Abstract

**Introduction:**

Intradural extramedullary (IDEM) spinal cord tumors account two thirds of all intraspinal tumors. These tumors produce pain syndromes, a variety of neurological symptoms-motor, sensory, sphincter or a combination of thereof. Preferred treatment is microsurgical radical resection. The objective of this study was analyzed and discuss about functional outcome in IDEM.

**Presentation of case:**

We present serial case of 15 patients, that consist of 8 males and 7 females, with the mean age of 43,4 years old, ranging from 16 to 82 years old. The outcomes were followed up with Karnofsky Score and Tomita to analyzed metastasis of the tumor. The mean of Karnofsky score in this study was 74 with no patient had metastatic intradural tumor, therefore the Tomita score in these patients is incalculable.

**Discussion:**

MRI confirmed the location and extent of the tumor for definitive diagnosis. We then performed excision of the tumor or decompression of the spinal canal followed by posterior stabilization if needed. Minimal complaint regarding pain and/or numbness were found. Post operation functional outcome of the patient is monitored using Karnofsky score.

**Conclusion:**

In all except one patient, the functional outcome of the patients is greater than 50 based on Karnofsky score. In this case series, the good functional outcome is due to all tumor were primary tumor which originate from the spine. Furthermore, all tumor had benign characteristic based on the anatomical pathology result. In consequence, gross total resection can be achieved thus resulting a good overall functional outcome of the patients.

## Highlights

• 14 of 15 patients had Karnofsky score of above 50.
• All tumor had benign characteristic based on the anatomical pathology result.
• Gross total resection can be achieved thus resulting a good overall functional outcome of the patient.

## 1 Introduction

Intradural extramedullary (IDEM) spinal tumors account for 40%–60% of intraspinal tumors and mainly represented by nerve sheath tumors and meningiomas. These two tumors cover about 55% of IDEM [1] tumors. Other tumors of the spine are schwannomas (30%; incidence rate, 0.3–0.4 cases annually per 100,000 people), meningiomas (25%; incidence rate, 0.32 cases annually per 100,000 people), neurofibromas, teratomas, lipomas, and metastatic tumors [2].

All of these tumors are unique. They produce pain syndromes, a variety of neurological symptoms-motor, sensory, sphincter or a combination thereof. All spinal levels may be involved. The diagnostics include magnetic resonance imaging (MRI) with contrast enhancement, computerizing tomography (CT) scanning (bone windows with reconstruction) and possibly CT myelograms. Preferred treatment is the microsurgical radical resection [3].

The aim of this study is to analyze and discuss about functional outcome in intradural extramedullary tumor in 15 patients. This paper has been written according to the PROCESS guideline [4].

### 1.1 Presentation of Case

We present serial case of 15 IDEM patients, were collected from 2015 until 2017, consisting of 8 males and 7 females, with the mean age of 43,4 years old (16–82 years). Major chief complain was pain and paresthesia. Neurologic involvement was found in 5 patients. Most of the patients has Frankle E or D. The highest Karnofsky score in this study was 90 and the lowest Karnofsky score in this study was 0 (Table 1).

**Table 1:** Demographics, diagnosis, and functional outcome of the presented cases.

Name (initial)	Age (years old)	Gender	Diagnosis	Procedure	Anatomical pathology	Karnofsky Score	SF-36
Patient 1	62	F	L4	Biopsy	Inflammatory tissue	60	without metastasis
Patient 2	63	F	L2	Excision and posterior stabilization	Schwannoma	90	without metastasis
Patient 3	28	M	Cauda Equida Tumor	Excision and posterior stabilization	Schwannoma	90	without metastasis
Patient 4	33	M	Brown Tumor Regio Thoracolumbar	Excision, and posterior stabilization	Schwannoma	80	without metastasis
Patient 5	50	F	Intradural Tumor	Excision, and posterior stabilization	Unknown	90	without metastasis
Patient 6	82	M	Paraparese inferior due to intradural tumor	Decompression, posterior stabilization	Benign cyst	50	without metastasis
Patient 7	47	F	Th1-2	Decompression, posterior stabilization	Unknown	80	without metastasis
Patient 8	16	M	Intrapeksi tumor	Biopsy	Hemangioma	0	without metastasis
Patient 9	37	F	Th 12	Biopsy, Excision, posterior stabilization	Conception tissue	80	without metastasis
Patient 10	21	M	Th 12	Excision, posterior stabilization	Schwannoma	70	without metastasis
Patient 11	54	M	L1	Decompression, posterior stabilization	Schwannoma	90	without metastasis
Patient 12	32	F	T6-7	Decompression, excision, posterior stabilization	Schwannoma	90	without metastasis
Patient 13	61	F	L1	Decompression, posterior stabilization	Benign cyst	60	without metastasis
Patient 14	10	M	T12 – L3	Excision	Schwannoma	90	without metastasis
Patient 15	55	M	Primary bone tumor of the T4	Biopsy, decompression, posterior stabilization	Schwannoma	90	without metastasis
**Mean**	**43,4**					**74**	

MRI confirmed the location and extent of the tumor to determine the diagnosis and the major course of the treatment. Mean size of the tumors were 4 cm and there were no extradural involvement.

The procedure was performed by spine consultant and also senior orthopedic resident. We then performed excision of the tumor or decompression of the spinal canal followed by posterior stabilization if needed. We did decompression of the spinal cord if we found neurological deficit from these patients and the spinal cord compression was indicated. The posterior stabilization procedures were done in 12 patients whom undergone excision or decompression that involved structural stability. There was no complication during the operative procedure.

The resected tissue then was sent to the pathologic department to be analyzed. No complication such as infection, neurovascular disturbance was found. Patients were discharged 1 week after the operation. Post operation functional outcome of the patient was monitored using Karnofsky score.

## 2 Discussion

### 2.1 Procedure

The surgical resection of a spinal cord tumor has been performed through total laminectomy. This facilitates access and visualization. Seppälä MT et al., reported a series of 187 patients that underwent surgical resection for spinal schwannoma and reported satisfactory prognoses. In this series, 62,5% were resected, with a 37,5% surgical complication rate [5]. However, total laminectomy may cause spinal instability and kyphosis due to the damage to the musculoligamentous structures and posterior bony elements. These complications may produce neurologic symptoms by compressing the spinal cord or nerve roots. In order to prevent such complications, a total laminectomy with arthrodesis or a unilateral limited laminectomy is needed. It may be assumed that the view is narrow in a unilateral laminectomy, so it is not easy to handle the instruments and the exposure of the lesion is also incomplete. As a result, normal nerves may be damaged or it may be difficult to remove the lesion completely. The main goal of treatment of IDEM spinal tumors is to do radical resection without mortality and minimal perioperative morbidity. Thorough perioperative planning, meticulous microsurgical techniques and early mobilization and rehabilitation are essential for good clinical outcomes [1].

### 2.2 Outcome

The Karnofsky Performance Status (KPS) was originally developed to document physical function and need for assistance in cancer patients, and it was shown to have very good interrater reliability. It can be used by the clinician as a cue to areas in which patients may be having problems and may be in need of rehabilitation [6]. The mean KPS of our patient is 74 which means there were evidence of severe disease, severe difficulty of daily activities, moderate difficulty of self-care grooming and severe work-difficulty.

Bennett et al. reported the KPS of an intradural extramedullary metastatic small cell lung cancer lesion to the cervical spine patient who underwent successful stereotactic radiosurgery (SRS) was 70 [7]. A patient who underwent minimal invasive approach for cervical schwannoma in the other study by Kumar et al. has 80 Karnofsky score (KPS) [8]. A retrospective case series was carried out with a patient sample of 82 male and female patients with non-syndromic spinal schwannomas and reporting the mean KPS in 1 year follow-up as 88,18 [9]. Thus, our study has worse Karnofsky Performance Score (KPS) result with the previous studies according to the mean score.

### 2.3 Complication

No complications of infection, neurovascular, non-union nor joint stiffness were found. The result was similar to a large series (367 cases) by Lenzi et al. [10]. This study had a median follow-up of 10 years (range 1–20) reported full or partial recovery in the great majority of patients with root pain resolving in all but nine patients. Another study also showed that most type of intradural tumor have 5-year survival rate >65%, this value was true except for intradural-extramedullary metastases which has mean survival time of 5–7,3 months [11,12]. In conclusion intradural tumor have great prognosis if the tumor is resected effectively.

### 2.4 Anatomical pathology

Intradural schwannoma is one of the most common form of intradural tumors. This tumor belongs to the nerve sheath tumor group. In one study, the prevalence is as high as 33% of the sampel population [13,14]. This is also fit the data form this study: 8 out of 15 patients had intradural tumor which can be categorized as schwannoma. Other study also showed that the anatomical pathology result were schwannomas (33%), meningiomas (22%), ependymomas (12%), and other pathologies (20%); pathology was unknown in 13% of patients [13].

## 3 Conclusion

In all except one patient, the functional outcome of the patient is greater than 50 based on Karnofsky score. In this case series, the good functional outcome is due to all tumor were primary tumor which originate from the spine. Furthermore, all tumor had benign characteristic based on the anatomical pathology result. In consequence, gross total resection can be achieved thus resulting a good overall functional outcome of the patient. Further study is needed with bigger sample as it is our limitation in this study.

## Ethical approval

This is a case series; therefore, it did not require ethical approval from ethics committee. However, we have got permission from the patients to publish his data.

## Sources of funding

No sponsorship for this case series.

## Author contribution

Fachrisal contributed in performing the surgical procedure, data collection, data analysis and writing the paper.

Eko Setiawan contributed in data collection, data analysis and writing the paper.

Sammy S. Alhuraiby contributed in data collection, data analysis and writing the paper.

## Registration of research studies

Name of the registry: http://www.researchregistry.com.

Unique Identifying number or registration ID: researchregistry5283.

Hyperlink to the registration (must be publicly accessible): https://www.researchregistry.com/browse-the-registry#home/registrationdetails/5df850f29e17330015034f9b/

## Guarantor

The guarantor is Fachrisal, M.D.

## Ethical Clearance

Ethical clearance was not required for this case series. This series has been registered in the Research Registry website, UIN researchregistry5283.

## Provenance and peer review

Not commissioned, externally peer reviewed.

## Declaration of competing interest

The authors declare that there is no conflict of interest regarding publication of this paper.

## References

[1] Hospital B., Sharma G.R., Hospital B. (2016).

[2] Govind M., Radheyshyam M., Achal S., Ashok G. (2016).

[3] Arnautovic K, Arnautovic A. Kenan arnautovic*, Aska Arnautovic. 9(Supplement 1):40–45.

[4] Agha R.A., Borrelli M.R., Farwana R., Koshy K., Fowler A.J., Orgill D.P. (2018). The PROCESS 2018 statement: updating consensus preferred reporting of CasE series in surgery (PROCESS) guidelines. Int. J. Surg..

[5] Seppälä M.T., Haltia M.J., Sankila R.J., Jääskeläinen J.E., Heiskanen O. (1995 Oct). Long-term outcome after removal of spinal schwannoma: a clinicopathological study of 187 cases. J. Neurosurg..

[6] Schag C.C., Heinrich R.L., Ganz P.A. (1984). Karnofsky performance status revisited: reliability, validity, and guidelines. J. Clin. Oncol..

[7] Bennett E.E., Berriochoa C., Habboub G., Brigeman S., Chao S.T., Angelov L. (2017). Rapid and complete radiological resolution of an intradural cervical cord lung cancer metastasis treated with spinal stereotactic radiosurgery: case report. Neurosurg. Focus.

[8] Kumar R., Dhal A.C., Kumar A. (2017). Case report: minimal invasive approach in a case of cervical schwannoma. Rom Neurosurg.

[9] Sun I., Pamir M.N. (2017). Non-syndromic spinal schwannomas: a novel classification. Front. Neurol..

[10] Ottenhausen M., Ntoulias G., Bodhinayake I., Ruppert F., Schreiber S. (2018). Intradural spinal tumors in adults — update on management and outcome. Neurosurg. Rev..

[11] Rinaldo L., McCutcheon B.A., Kerezoudis P., Shin J.H., Mehta A.I., Clarke M.J. (2016). Outcomes after surgical treatment of intradural-extramedullary spinal cord tumors: a review. World Spinal Column J.

[12] Halvorsen C.M., Rønning P., Hald J., Johannesen T.B., Kolstad F., Langmoen I.A. (2015). The long-term outcome after resection of intraspinal nerve sheath tumors: report of 131 consecutive cases. Neurosurgery.

[13] Korn A., Halevi D., Lidar Z., Biron T., Ekstein P., Constantini S. (2015). Intraoperative neurophysiological monitoring during resection of intradural extramedullary spinal cord tumors: experience with 100 cases. Acta Neurochir..

[14] Turel M.K., D'Souza W.P., Rajshekhar V. (2015). Hemilaminectomy approach for intradural extramedullary spinal tumors: an analysis of 164 patients. Neurosurg. Focus.

