# Supplementary material for: Functional outcome in intradural extramedullary tumor patients: Case series
Source: Ann Med Surg (Lond). 2020 Apr 24;54:71–3. doi: 10.1016/j.amsu.2020.04.009 (PMC7201154; doi:10.1016/j.amsu.2020.04.009)
Supplement: Multimedia component 1 [file mmc1.docx]

| PROCESS 2018 Checklist | | | |
| --- | --- | --- | --- |
| Section | **Item** | **Checklist Description** | **Page Number** |
| Title | 1 | Both the words “case series” and the area of focus should appear in the title (e.g. disease, exposure/intervention or outcome) | 1 |
| Abstract | 2a | Introduction - what is the unifying theme of the case series. | 1 |
|  | 2b | Methods - describe what was done, how and when was it done and by whom. |  |
|  | 2c | Results - what was found. |  |
|  | 2d | Conclusion - what have we learned and what does it mean |  |
| Introduction | 3 | Background and relevance - Explain the scientific background and rationale for the case series (e.g. specify the unifying theme - common disease, exposure, intervention and outcome). The introduction should explain why this study needed. | 2 |
| Methods | 4a | Registration - state the research registry number in accordance with the declaration of Helsinki - "Every research study involving human subjects must be registered in a publicly accessible database" (this can be obtained from; ResearchRegistry.com or ClinicalTrials.gov or ISRCTN). If a protocol exists already, state where it can be accessed (must be publicly accessible). | 4 |
|  | 4b | Study design - state the study is a case series. In addition, it is necessary to state whether the case series is: 1) prospective or retrospective in design; 2) single or multi-centre; and 3) cases are consecutive or non-consecutive. | 2 |
|  | 4c | Setting - describe the setting(s)and nature of the institution in which the patient was managed; academic, community or private practice setting? Location(s), and relevant dates, including periods of recruitment, exposure, follow-up, and data collection | 2 |
|  | 4d | Participants - describe the relevant characteristics of the participants (comorbidities, tumour staging, smoking status, etc). State any eligibility (inclusion/exclusion) criteria and the sources and methods of selection of participants. Describe length and methods of follow-up. | 2 |
|  | 4e | Pre-intervention considerations e.g. Patient optimisation: measures taken prior to surgery or other intervention e.g. treating hypothermia/hypovolaemia/hypotension in burns patients, ICU care for sepsis, dealing with anticoagulation/other medications and so on. | 2 |
|  | 4f | Types of intervention(s) deployed (pharmacological, surgical, physiotherapy, psychological, preventive) and reasoning behind treatment offered. | 2 |
|  | 4g | Intervention details – details on how the intervention was carried out. For surgery, for example, include information on anaesthesia, patient position, use of tourniquet and other relevant equipment, preparation used, sutures, devices, surgical stage (1 or 2 stage, etc). For pharmacological therapies, include formulation, dosage, strength, route and duration. | 2 |
|  | 4h | Who performed the procedures – the operator position and their experience experience (position on the learning curve for the technique if established, specialisation and prior relevant training). For example, ‘A junior resident, three years into specialized training’. Degree of novelty for a surgical technique/device should be mentioned and a comment on learning curves should be made for new techniques/devices. | 2 |
|  | 4i | Quality control - what measures were taken to reduce inter or intra-operator variation, ensure quality, and maintain consistency between each case in the delivery of the intervention e.g. independent observers, lymph node counts, standard surgical technique. | 2 |
|  | 4j | Post-intervention considerations – following the main intervention: 1) when were the patients followed-up; 2) where; 3) what did follow-up entail (additional tests, scans, clinical examination) and what were the results of these; and 4) were there any post-operative instructions. | 2 |
| Results | 5a | Participants - reports numbers involved and their characteristics (including, most importantly, their comorbidities and smoking status, as well as other demographic details). For all cancer patients it is necessary to include details on tumour staging (e.g. TNM) | 3 |
|  | 5b | Changes to reports – report any changes in the interventions during the course of the case series (what the change was, reasons for the change, what learning occurred, together with rationale and a diagram if appropriate). | 3 |
|  | 5c | Outcomes and follow-up - Clinician assessed and patient-reported outcomes (when appropriate, including, for example questionnaires or comments at outpatient visits) should be stated. Include details on the time periods at which assessed. Relevant photographs/radiological images should be provided e.g. 12 month follow-up. Describe loss to follow-up (express as a percentage) and any explanations for it. | 3 |
|  | 5d | Intervention adherence/compliance - where relevant how well patients adhered to and tolerated their treatment. For example, post-operative advice (heavy lifting for abdominal surgery) or tolerance of chemotherapy and pharmacological agents. | 3 |
|  | 5e | Adverse events – all complications and adverse or unanticipated events should be described in detail and ideally categorised in accordance with the Clavien-Dindo Classification. How they were prevented, diagnosed and managed. Blood loss, operative time, wound complications, re-exploration/revision surgery, 30-day post-op and long-term morbidity/mortality may need to be specified. If there were no complications or adverse outcomes this should also be included. | 2 |
| Discussion | 6a | Summarise key results | 4 |
|  | 6b | Placing results in context – describe all relevant literature, describe the prevailing gold standard should one exist, and describe how findings reported compare with established therapies. State the implications for clinical practice guidelines and any relevant hypotheses that have been generated as a result of this worth | 4 |
|  | 6c | Strengths and limitations of the study | 4 |
|  | 6d | Future – State the further research that can be done to build on the findings and methodology discussed. State the study design next best suited to address these areas. | 4 |
|  | 6e | Rational – ensure any conclusions made have strong rationale | 4 |
| Conclusions | 7a | State the key conclusions from the study | 4 |
|  | 7b | State what needs to be done next, further research with what study design. | 4 |
| Additional Information | 8a | State any conflicts of interest | 4 |
|  | 8b | State any sources of funding | 4 |
|  | 8c | State Ethics - state whether ethical approval was needed and if so, what the relevant judgement reference was? | 4 |
